# Gut microbiome modulation in allergic rhinitis: from current evidence to emerging therapies

**DOI:** 10.3389/falgy.2026.1761840

**Published:** 2026-04-01

**Authors:** Siu-Jung Au Yong, Amanda Sandra Lestari Lee, Vetriselvan Subramaniyan, Chiau Ming Long, Salina Husain, Kavita Reginald, Hooi-Leng Ser

**Affiliations:** 1 Department of Biomedical Sciences, Jeffrey Cheah Sunway Medical School, Faculty of Medical and Life Sciences, Sunway University, Bandar Sunway, Malaysia; 2 School of Pharmacy, Sunway University, Bandar Sunway, Malaysia; 3 Department of Otorhinolaryngology-Head and Neck Surgery, Faculty of Medicine, Universiti Kebangsaan Malaysia, Kuala Lumpur, Malaysia

**Keywords:** allergic rhinitis, gut-airway axis, immune tolerance, microbial dysbiosis, microbiome-based therapeutics

## Abstract

Allergic rhinitis (AR) is a common inflammatory disorder of the upper airway that is primarily managed with pharmacotherapy, biologics and allergen immunotherapy. However, a substantial proportion of patients experience incomplete or insufficient symptom control, treatment-related adverse effects, or poor adherence. Increasing evidence has linked AR with alterations in microbial composition across multiple mucosal sites, including the gut, highlighting potential roles for host-microbiome interactions in the regulation of allergic inflammation, although causal relationships remain incompletely defined. This narrative mini-review synthesizes current evidence on gut microbiome-based interventions for allergic rhinitis (AR), including probiotics, prebiotics, synbiotics, postbiotics, and emerging approaches such as fecal microbiota transplantation, engineered microbes, and bacteriophage-based therapies. It examines proposed immunological mechanisms involving type 2 inflammation, regulatory immune pathways, and gut–airwa*y* axis signalling, while distinguishing clinically evaluated strategies from experimental or preclinical and assessing their translational readiness. Collectively, available evidence suggests that microbiome-targeted therapies represent a promising conceptual avenue for understanding and potentially modulating AR. However, their clinical application remains constrained by heterogeneous study designs, reliance on extrapolated data from preclinical studies, limited standardized outcome measures, insufficient long-term safety data, and evolving regulatory frameworks. Addressing these challenges through well-designed clinical trials and improved mechanistic characterization will be essential to clarify the role of microbiome-based interventions as adjunctive strategies in AR management.

## 1 Introduction

Allergic rhinitis (AR) is a common chronic inflammatory disorder of the upper airways, characterized by IgE-mediated immune responses to environmental allergens. Its reported prevalence varies substantially depending on geographical region, population, diagnostic criteria, and study design, with large epidemiological studies indicating adult prevalence estimates most commonly between 10% and 20%, and pediatric estimates ranging from 5%–25% (1, 2). Although not life-threatening, AR significantly impairs quality of life, contributing to sleep disturbances, reduced school and work productivity, and substantial socio-economic burden (3).

Current management strategies, including antihistamines, intranasal corticosteroids, and allergen immunotherapy (AIT), provide effective symptomatic relief for many patients. However, real-world evidence indicates that more than one-third patients continue to experience inadequate symptom control, treatment-related adverse effects or poor adherence (4–6). In addition, AIT requires prolonged treatment courses and demonstrates variable patient responsiveness. These limitations highlight the need to better understand the underlying mechanisms of AR and to identify novel therapeutic strategies that may provide more sustained disease control (7).

In recent years, growing attention has focused on the role of the human microbiome in immune regulation and allergic diseases (8). Advances in high-throughput sequencing have revealed that alterations in microbial communities across multiple mucosal sites, including the gut, may influence host immune responses and contribute to allergic inflammation (9–11). In particular, the gut microbiome plays an essential role in shaping host immune homeostasis, including the development of regulatory T cells (Tregs), modulation of inflammatory pathways, and maintenance of epithelial barrier integrity (12, 13). These interactions have led to the concept of the gut-airwa*y* axis, whereby gut-derived microbial metabolites and immune signals can influence immune responses at distant mucosal sites, including the respiratory tract (14). Through this axis, alterations in gut microbial composition may contribute to the development or exacerbation of airway inflammatory diseases, including AR.

Recent microbiome studies have begun to characterize the general patterns of gut dysbiosis associated with AR. Although specific microbial taxa vary across studies and populations, several recurring features have been reported. Individuals with AR frequently exhibit reduced gut microbial diversity together with alterations in the relative abundance of bacterial taxa involved in short-chain fatty acid (SCFA) metabolism (15, 16). In particular, decreased representation of SCFA-producing bacteria such as *Faecalibacterium*, *Roseburia*, *Blautia*, and *Eubacterium* has been reported in some cohorts, which may contribute to reduced production of butyrate and other immunomodulatory metabolites. These metabolites are known to promote Treg differentiation, strengthen epithelial barrier integrity, and suppress type 2 inflammatory responses (16). Conversely, enrichment of potentially pro-inflammatory taxa including *Bacteroides*, *Prevotella*, and *Escherichia-Shigella* has been reported in certain populations (9, 15, 16). Collectively, these compositional and functional shifts suggest that disruption of microbial metabolic functions may contribute to impaired immune tolerance and Th2-skewed inflammation in AR.

Given the emerging evidence linking gut microbial dysbiosis with allergic inflammation, increasing attention has focused on microbiome modulating strategies aimed at restoring microbial balance and immune homeostasis. These approaches include probiotics, prebiotics, synbiotics, and postbiotics, as well as emerging strategies such as fecal microbiota transplantation (FMT) and engineered microbial therapies. However, the evidence supporting these interventions remains heterogenous and translational readiness varies considerably (17). This narrative mini-review provides a critical synthesis of current evidence on gut microbiome-based interventions in AR. We evaluate established and emerging microbiome-modulating strategies, examine their proposed immunological mechanisms within the gut-airwa*y* axis, and assess their translational potential and current limitations in clinical practice.

## 2 Current conventional therapies for AR and their limitations

Conventional pharmacotherapies for AR, including antihistamines, intranasal corticosteroids (INS), decongestants, and leukotriene receptor antagonists, primarily provide symptomatic relief rather than sustained disease control. Real-world evidence indicates that a substantial proportion of patients experience incomplete symptom control, recurrence of symptoms upon treatment discontinuation, treatment related adverse effects, or suboptimal adherence, limiting long-term effectiveness (18, 19).

Antihistamines are effective in alleviating sneezing, rhinorrhea, and nasal itching but are less effective in relieving nasal obstruction, which is often the most dominant and burdensome symptom of allergic rhinitis. First generation agents are associated with sedation and cognitive impairment, whereas second-generation agents, although better tolerated, still fail to adequately address congestion in many patients (19). INS remain the most effective anti-inflammatory treatment for AR. In a meta-analysis synthesizing data from five randomized controlled trials involving 990 patients, Juel-Berg et al. demonstrated superior efficacy of INS compared with oral antihistamines in reducing total nasal symptom scores (20). Despite their effectiveness, long-term INS use is commonly limited by local adverse effects such as epistaxis, nasal irritation, and discomfort, as well as incorrect administration technique; all of which negatively affect adherence in routine clinical practice (21). Decongestants offer rapid but short-lived relief of nasal obstruction, but prolonged topical use is associated with rhinitis medicamentosa, while systemic formulations may cause cardiovascular adverse effects, restricting their use in susceptible populations (22). Leukotriene receptor antagonists may confer modest benefit for nasal obstruction but are generally less effective than INS for overall disease control and rarely achieve adequate symptom relief as monotherapy (23). More recently, biologic therapies targeting type 2 inflammatory pathways, including omalizumab, mepolizumab, and dupilumab, have shown efficacy in selected patients with severe or refractory disease (24). Despite that, their use in AR remains limited by high cost, parenteral administration, restricted indications, and the need for specialist care (25).

AIT is the only intervention capable of modifying the natural course of AR through the induction of immune tolerance. Nevertheless, its clinical utility is constrained by the requirement for prolonged treatment over several years, variable patient responsiveness, adherence challenges, and the risk of adverse reactions, ranging from local reactions to rare systemic events (26, 27). Furthermore, the absence of robust predictive biomarkers limits optimal patient selection and treatment individualization (27).

Collectively, these limitations underscore persistent gaps in current AR management, particularly with respect to achieving durable remission, minimizing adverse effects, and improving long-term adherence. These unmet needs provide a clear rationale for exploiting adjunctive or complementary approaches, including microbiome-based strategies, to support more sustained and patient-tailored disease control.

## 3 Pathophysiology of microbial dysbiosis and the gut-airwa*y* axis in AR

The gut microbiome, consisting of trillions of microorganisms including bacteria, viruses, fungi, and archaea resides in the gastrointestinal tract and plays a vital role in maintaining health and contributing to disease The maturation of the gut microbiome and immune system begins at birth and reaches a stable state by the age of three, with early microbial colonisation being critical for immune tolerance to benign antigens and protection against pathogens (28). The eubiotic state or “eubiosis,” refers to the desired state of host gut health in terms of a balance of microbial population. Intestinal epithelial cells (IECs) play a crucial role in maintaining intestinal homeostasis by regulating immune responses to gut microbiome metabolites, such as SCFAs (29). They also act as a physical barrier, preventing pathogens from entering systemic circulation. Gut dysbiosis is associated with the “leaky gut” phenomenon, characterised by increased intestinal permeability and heightened inflammation due to mucosal barrier dysfunction and consequently lead to the development of diseases, including AR (30).

In healthy individuals, the gut microbiome is dominated by Actinobacteria, Firmicutes, Proteobacteria and Bacteroidetes, which together account for approximately 90% of the microbial population (31, 32). These microbial communities maintain epithelial barrier integrity, regulate metabolic activity and promote immune tolerance through metabolites such as SCFAs. Conversely, several studies have reported a compositional shift in the gut microbiome of AR patients, including the reduction in Actinobacteria, Firmicutes, and Proteobacteria, accompanied by an increase in Bacteroidetes (33–36), suggesting disrupted immune homeostasis. A commonly reported indicator of microbial imbalance is the Bacteroidetes/Firmicutes (B/F) ratio, which has been proposed as a marker of dysbiosis associated with allergic disease development. In addition to phylum-level shifts, AR patients often exhibit enrichment of Gammaproteobacteria and Deltaproteobacteria, including pro-inflammatory genera such as *Escherichia–Shigella* and *Bilophila*, alongside reduced abundance of beneficial genera such as *Alistipes* and *Prevotella* compared with healthy individuals (33, 37–39). Beyond compositional changes, gut dysbiosis may also affect the functional metabolic capacity of the microbiome. AR-associated dysbiosis is frequently characterized by reduced abundance of SCFA-producing bacteria, including *Faecalibacterium*, *Bifidobacterium, Alistipes* and *Prevotella* (10, 40). These metabolites contribute to epithelial barrier integrity and promote Treg differentiation, supporting immune tolerance and limiting excessive inflammatory responses (41). On top of that, the enrichment of pro-inflammatory taxa such as *Shigella* may enhance cytokine production, including interleukin-17 (IL-17), contributing to immune hyperactivation and allergic inflammation (42). These findings highlight the role of gut microbial composition in shaping immune responses and influencing allergic disease outcomes (43).

Apart from affecting the intestinal immune homeostasis, these microbial alterations may also influence distal mucosal sites, including the respiratory tract. Increasing evidence suggests that these microbial-immune interactions extend beyond the gastrointestinal tract through the gut–airwa*y* axis—a bidirectional communication network linking intestinal and respiratory immune environments (10, 44). Gut microbial-derived metabolites (including SCFAs, tryptophan metabolites, and bile acids) can circulate systemically and influence epithelial barrier function, inflammatory signaling and microbial composition within the respiratory tract. Recent studies have proposed that gut microbial-derived metabolites contribute to chronic inflammatory pathology in AR via the gut-airwa*y* axis, specifically phenylacetate–glutamine conjugates and trimethylamine N-oxide (TMAO) may promote systemic inflammation via increasing NLR family pyrin domain containing 3 (NLRP3) inflammasome and pattern-recognition receptors (e.g., toll-like receptor 2 (TLR2) and toll-like receptor 4 (TLR4)), while dysregulated tryptophan metabolism may weaken barrier maintenance which may eventually promote systemic inflammation (14, 16).

Consequently, considering the bidirectional nature of the gut–airwa*y* axis, it is plausible that alterations in the nasal microbiome may influence gut health and subsequently contribute to systemic inflammation. This bidirectional communication may be mediated in part by microbial extracellular vesicles, which serve as vehicles for transporting microbial metabolites and signaling molecules across mucosal sites, including the gut. In patients with AR, the nasal mucosa frequently exhibits reduced microbial diversity alongside an enrichment of opportunistic taxa such as *Staphylococcus*, *Klebsiella*, and *Moraxella*, while the abundance of protective commensal genera including *Corynebacterium* is diminished (45–47). Altered interactions between the nasal microbiota and local metabolome further contribute to shaping the inflammatory microenvironment. A recent study reported that the enrichment of the *Aeromonas* genus in AR patients was positively correlated with pro-inflammatory lysophosphatidylcholine (LPC) and inversely associated with anti-inflammatory metabolites, such as cnidioside A (11). In other words, an increased abundance of *Aeromonas* in AR patients may elevate LPC levels, which can exacerbate nasal inflammation and symptom severity. LPC is known to promote the recruitment and migration of immune cells through activation of G protein-coupled receptors, thereby further amplifying inflammatory responses in the nasal mucosa.

Beyond bacterial communities, gut virome and mycobiome are increasingly recognised as crucial components of gut health, regulating immune responses and maintaining gut homeostasis. Early-life alterations in the enteric DNA virome and bacteriophage communities are associated with atopic risk (48–50), including specific anellovirus compositions linked to AR development in childhood (51). Similarly, a study in 2021 revealed that respiratory viruses like human rhinovirus and human respiratory syncytial virus contributes to rhinitis and wheezing disorders in infants (52). Distinct mycobiome structures have been identified in the nasal vestibule where *Malassezia* species frequently predominate (53–56). Pérez-Losada et al. has conducted an extensive study investigating the association between nasal mycobiome in AR patients (with and without asthma) and healthy controls (55). Interestingly, the authors reported that the majority of the most abundant fungal genera (7/10) including *Malassezia, Alternaria, Cladosporium, Penicillium, Wallemia, Rhodotorula, Sporobolomyces, Naganishia, Vishniacozyma*, and *Filobasidium* differed significantly in the nasal cavity between these groups (*p* ≤ 0.049). These findings are consistent with an earlier study conducted in 2015, which also observed significantly greater fungal diversity in patients with AR compared with healthy individuals (53, 54). Such compositional shifts in the mycobiome have therefore been proposed as potential indicators and predictive markers of disease.

However, evidence regarding the roles of the virome and mycobiome in allergic diseases remain limited. Further studies are required to elucidate the mechanisms and functional contributions of specific viruses and fungi in driving inflammation and influencing disease severity in AR (57).

## 4 Current evidence for microbiome-based therapeutics in AR

**Figure 1:**
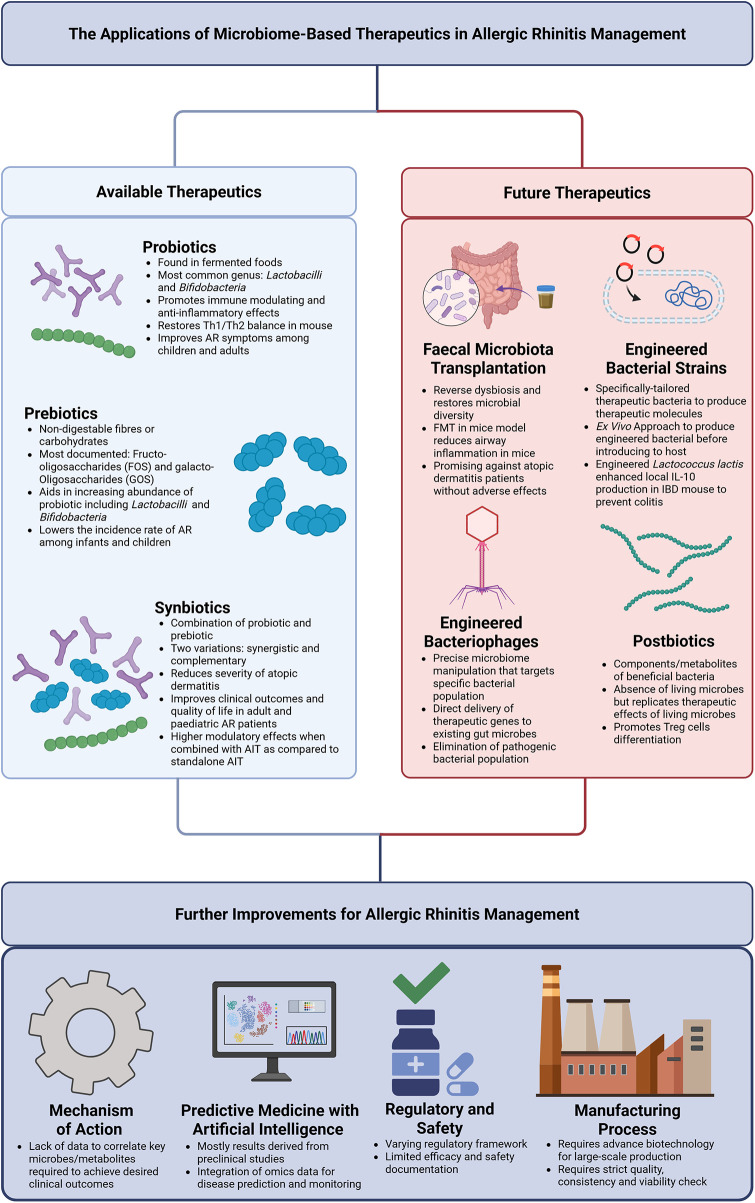
Schematic overview of microbiome-based therapeutic strategies proposed for allergic rhinitis.

Given the emerging evidence linking AR to alterations in gut microbial composition and function, considerable attention has focused on microbiome-modulating strategies aimed at restoring microbial balance. Evidence from both animal and human studies demonstrates that microbiome-based interventions, including probiotics, prebiotics, and synbiotics, hold potential to modulate immune responses and contribute to improved clinical outcomes in AR (Table 1, Figure 1). However, reported benefits vary substantially according to probiotic strain, dose, formulation, population characteristics, and study design. Accordingly, observed effects should be interpreted as strain and context-specific rather than representative of uniform probiotic efficacy. Representative clinical and preclinical examples illustrating these effects are summarized in Table 1.

**Table 1:** Effects of probiotics, prebiotics, and synbiotics on allergic rhinitis (AR) and related allergic outcomes in animal and human studies

Intervention type	Strain/Compound	Model/Population	Clinical outcomes	Immunological effects	Reference
Probiotic	*Lactobacillus plantarum*	Mouse AR model (BALB/c)	↓ Leukocyte infiltration, ↓ airway hyperreactivity	Restored Th1/Th2 balance	(59)
	*Lactobacillus rhamnosus* GG	Mouse AR model (A/J and C57BL/6)	–	↑ Regulatory *T* cells, ↑ CD103^+^ dendritic cells	(118)
	*Lactobacillus plantarum* CJLP133/243	Mouse AR model (BALB/c)	↓ Nasal/lung immune infiltration, ↓ Airway hyperreactivity, Improved AR symptoms	↑ IgG2a, ↑ Th1:Th2 ratio	(119)
	*Bifidobacterium longum* BB536	Adults with Japanese cedar pollen allergy	↓ Rhinorrhoea, ↓ Nasal blockage, ↓ Medication use	↑ Bifidobacteria, ↑ Th1 cytokines, suppression of *B. fragilis*	(120–122)
	*Lactobacillus plantarum* GUANKE	Adults with AR	↓ Serum IgE, ↓ Symptom severity	Improved Th1/Th2 balance	(123)
	*Lactobacillus paracasei* LP-33	Children <5 years	81% complete symptom relief, comparable to cetirizine	–	(61)
	*L. paracasei* HF.A00232 + levocetirizine	Children 6–13 years	Improved symptoms (sneezing, nasal irritation, puffy eyes) after antihistamine withdrawal	Improved PRQLQ^a^ scores	(78)
	*L. rhamnosus* HN001 (maternal); *B. lactis* HN019	Infants (maternal supplementation, and from birth to age 2 years in infants)	↓ Risk of rhinitis in offspring (HN001 only)	–	(62)
	Multi-strain (*L. acidophilus*, *L. delbrueckii*, *L. rhamnosus*, *S. thermophilus*)	Adults with AR	↓ Symptoms, ↑ quality of life, ↑ microbiome diversity	↓ Eosinophils, basophils, inflammatory cytokines	(69)
	*L. rhamnosus* GG + *L. gasseri* TMC0356	Adults with AR/atopic dermatitis	↓ AR and AD symptoms vs placebo	Modulated gut microbiota composition	(124, 125)
	*L. paracasei* NCC2461 (single strain) vs blends of of *L. acidophilus* ATCC SD5221 and *B. lactis* ATCC SD5219	Adults with grass pollen allergy	↓ Nasal pruritus (NCC2461), no effect with blends	↓ Nasal leukocytes	(68)
Prebiotic	Short-chain galacto-oligosaccharides/long-chain fructo-oligosaccharides	Infants (hypoallergenic formula)	↓ Incidence of AR and rhinoconjunctivitis; long-term protective effect	↑ *Bifidobacteria*, ↑ *Lactobacilli*	(70, 71)
	Polydextrose and galacto-oligosaccharides	Children (cow's milk formula)	↓ Allergic attacks, ↓ AR incidence (Hazard ratio = 0.64, p = 0.007)	–	(72)
Synbiotic	Multi-strain probiotics + fructo-oligosaccharides	AR patients	Improved SNOT-22^b^, CARAT^c^, and SF-36^d^ scores	↑ IFN-γ, TGF-β, FoxP3; ↓ IL-4, IL-13, IL-17	(75, 76)
	*L. acidophilus* Rosell-52, *B. infantis* Rosell-33, *B. bifidum* Rosell-71 + Fructo-oligosaccharides+vitamin C	Children	↓ VAS^e^, ↓ TNSS^f^, ↑ RCAT^g^, ↑ PRQLQ^a^ vs placebo	–	(77)

^a^ PRQLQ, pediatric rhinoconjunctivitis quality of life questionnaire

^b^ SNOT-22, sino-nasal outcome test-22

^c^ CARAT, control of allergic rhinitis and asthma test

^d^ SF-36, short form 36-item health survey

^e^ VAS, visual analogous scale

^f^ TNSS, total nasal symptom score

^g^ RCAT, rhinitis control assessment test

In recent decades, probiotics have been extensively investigated in AR, with heterogeneous outcomes reported across studies. Certain well-characterized strains, including *Lactobacillus plantarum* (119, 123), *L. rhamnosus* (118) and *Bifidobacterium longum* BB536 (120–122), have been reported in some animal and human studies to improve in symptom scores, reductions in allergen-specific serum IgE, and modulation of T-helper 1/T-helper 2 (Th1/Th2) balance (58, 59). Mechanistic studies suggest that such effects may involve modulation of dendritic cell maturation, enhancement of regulatory T-cell responses, and alterations in gut microbiome composition; however, these mechanisms are not uniformly observed across strains or clinical settings (60).

In pediatric populations, some clinical trials have reported symptom improvements with specific probiotic strains. For instance, *L. paracasei* LP-33 demonstrated symptom relief comparable to cetirizine in one study, with a high proportion of children (81%) achieving symptom resolution (61). Additionally, evidence from early-life intervention studies indicates that both maternal (during pregnancy and 6 months postpartum) and infants (from birth and up to 11 years old) supplementation with *L. rhamnosus* HN001 is associated with a reduced risk of rhinitis in offspring, but not *Bifidobacterium lactis* HN019 (62). From these observations, it appears that early-life probiotic interventions are influenced by multiple factors, including strain selection and timing of exposure, which warrants further investigation to clarify their role in preventing atopic dermatitis (AD). The overall certainty of evidence for early-life probiotic interventions remains limited, largely due to heterogeneity in study design, strain selection, dosing regimens, and outcome measures (63, 64). Despite promising strain-specific findings, overall preventive efficacy remains uncertain when evaluated across broader meta-analyses encompassing heterogeneous populations and probiotic formulations. While the HN001 strain demonstrates long-term protective effects up to age 11 (62), large-scale meta-analyses report no statistically significant reduction in AR risk following probiotic use in pregnancy or infancy, highlighting that preventative efficacy is highly strain-specific and lacks clinical robustness (65–67).

On the other hand, several studies involving adult patients evaluating multi-strain probiotic formulations have reported improvements in symptom severity and quality-of-life measures (124, 125). Notably, head-to-head comparisons indicate that single strain formulations can achieve efficacy comparable to multi-strain products, highlighting that clinical benefit depends on strain selection rather than strain number (68, 69). Torre et al. discussed that probiotic-treated patients (for 60 days) displayed a significant decrease in Average Rhinitis Total Symptom Score, along with reduction in percentage of activated eosinophils and basophils. There was also a correlation between recovery of microbial diversity post probiotics treatment with reduction in the Th2 cytokine profile indicated by decreased levels of serum IL-4 and IL-5. On the contrary, Perrin et al. reported decreased in IL-5 secretion in nasal fluid of patients receiving single probiotic strain but not in patients who had taken a blend of *Lactobacillus acidophilus* ATCC SD5221 and *B. lactis* ATCC SD5219. Nevertheless, variability in trial design and outcomes measures limits direct comparability across studies.

Compared with probiotics, evidence for prebiotics in AR remains limited but suggestive. Supplementation with fructo-oligosaccharides (FOS), galacto-oligosaccharides (GOS), and polydextrose (PDX) has been associated in some infant and pediatric studies with reduced AR incidence and allergic manifestations, potentially mediated through enrichment of bifidobacteria and lactobacilli populations (70–72). In one study, the protective effect of combined short-chain GOS and long-chain FOS persisted until five years of age (71). In fact, the primary rationale for prebiotic administration is to selectively promote the growth and metabolic activity of health-associated microbes, thereby supporting microbial balance and immune homeostasis (73). While there is lack of study demonstrating the effectiveness of prebiotics in AR, researchers have also argued that the effects of prebiotics may depend on the presence, abundance, and functional capacity of specific microbial strains within the host microbiome (74). Consequently, increasing attention has been directed toward synbiotic strategies, in which defined prebiotics are paired with complementary probiotic strains to support microbial engraftment and functional activity.

Synbiotics have been proposed as a means of achieving additive or synergistic effects, although reported clinical outcomes involving AR patients to date remain heterogeneous. Several clinical trials have demonstrated improvements in symptom scores and quality of life compared with placebo or standard therapy, particularly when synbiotics are used as adjunctive interventions (75–77). In a placebo-controlled, randomized, double-blind clinical trial, synbiotic supplementation was associated with significant improvements in SNOT-22, CARAT, and SF-36 scores after two months of intervention (76). From an immunological perspective, synbiotic administration has been most consistently associated with attenuation of pro-inflammatory responses, particularly suppression of Th17-associated pathways and reduced IL-17 expression. In contrast, effects on cytokines involved in immune regulation and Th1/Th2 balance, such as interferon-gamma (IFN-γ), as well as regulatory mediators including transforming growth factor beta (TGF-β), appear variable and are not uniformly observed across studies (68, 76, 78).

Taken together, the current body of evidence suggests that microbiome-based strategies may have a role as adjunctive approaches in the management of AR. Probiotics remain the most extensively studied intervention, with available data indicating that observed effects vary according to strain and clinical context. In comparison, prebiotics and synbiotics are less well explored, with existing studies limited in number and characterized by heterogeneity in design and outcomes. Further well-designed clinical studies, incorporating consistent outcome measures and exploratory mechanistic assessments, will be important for improving understanding of the potential clinical relevance of these interventions and the pathways through which they may improve AR symptoms and severity.

## 5 Emerging and experimental microbiome-based therapeutic strategies

Over the past decade, research has increasingly highlighted the role of gut microbiome dysbiosis in the development and progression of AR (9, 79). Restoring microbial diversity therefore holds therapeutic potential, which could be achieved through approaches such as fecal microbiota transplantation (FMT), engineered bacterial strains, bacteriophage-based therapies, and postbiotics (Figure 1).

### 5.1 Fecal Microbiota transplantation (FMT)

FMT has emerged as a potential strategy to restore gut microbial diversity by transferring fecal material from a healthy donor to a recipient with dysbiosis, thereby modulating host immune responses (80–82). Preclinical studies indicate that FMT can modulate allergic inflammation. In an experimental mouse model of AR, FMT attenuated nasal inflammation, eosinophil infiltration, Th2-associated cytokine responses, and IgE levels, effects linked to modulation of CD4^+^ T-cell responses and restoration of gut microbiome composition (83). Similarly, in murine models of food allergy, FMT reduced allergic manifestations and IgE-mediated immune responses through alterations in gut microbial communities and associated immunoregulatory pathways (84, 85).

In humans, however, clinical evidence for FMT in allergic disease remains extremely limited. Small studies in atopic dermatitis have reported improvements in disease severity and reduced corticosteroid use without major adverse effects (86), but these findings arise from a distinct atopic phenotype and cannot be directly extrapolated to AR. Consequently, the current evidence base should be regarded as hypothesis-generating rather than indicative of established clinical efficacy or translational readiness for AR. At present, only one registered interventional clinical trial is specifically evaluating FMT in AR (NCT06372184) using a randomized, double-blind, placebo-controlled design, with an estimated completion date in 2029.

### 5.2 Engineered microbes

Advances in Clustered Regularly Interspaced Short Palindromic Repeats-associated protein (CRISPR-Cas) systems, synthetic biology, and genome-editing have now enabled the development of engineered microorganisms designed to deliver defined immunomodulatory molecules *in vivo* (87). Current strategies primarily involve *ex vivo*-engineered microbes administered to the host, most commonly via the gastrointestinal tract, as well as engineered bacteriophages intended to modify resident microbial communities *in situ*. To date, the majority of supporting evidence for these approaches derives from experimental models of intestinal inflammation rather than allergic airway disease (88).

A well-established example is the use of genetically engineered *Lactococcus lactis* strains that secrete interleukin-10 (IL-10). In murine models of colitis, these organisms reduced intestinal inflammation through local enhancement of regulatory immune responses (89, 90). Although IL-10 is recognized as a key regulatory cytokine in allergic disease, including in the modulation of Th2 responses, these findings arise from a gastrointestinal inflammatory context and cannot be directly extrapolated to AR, where immune mechanisms, tissue architecture, and effector pathways differ substantially (91, 92).

Other engineered microbial systems further illustrate the conceptual potential of this approach. For instance, engineered *Saccharomyces cerevisiae* strains capable of producing short-chain fatty acids such as butyrate have been shown in pre-clinical models to alter gut microbial composition, promote the expansion of beneficial taxa, and enhance regulatory Treg differentiation (93). While microbial metabolites are increasingly recognized as important modulators of immune function, these observations remain indirect with respect to AR pathophysiology and are confined to experimental settings.

In summary, these studies should be interpreted as proof-of-concept for *in vivo* immunomodulatory delivery by engineered microbes. At present, however, the application of engineered bacteria to AR management remains speculative. Key challenges include achieving disease-relevant immune-modulation at distal mucosal sites such as the upper airway, ensuring controlled gene expression and functional stability in complex *in vivo* environments, and addressing safety concerns related to persistence, horizontal gene transfer, and long-term ecological effects (94). In addition, the regulatory framework for genetically modified microorganisms intended for therapeutic use in humans remains underdeveloped, with limited guidance regarding their approval, monitoring, and clearance (95, 96). Substantial pre-clinical and clinical investigation will therefore be required before engineered microbial therapies can be considered viable candidates for AR treatment.

### 5.3 Bacteriophage therapy

Bacteriophages, viruses that infect bacteria, can modulate microbiome composition and function (49). They are broadly classified into lytic and temperate (lysogenic) phages. Lytic phages can target and eliminate harmful bacteria that drive Th2 inflammation, thereby indirectly promoting SCFA-producing genera such as *Alistipes* and *Prevotella* (97). However, bacterial lysis may release toxins, which could potentially trigger inflammatory responses. To mitigate this issue, genetically engineered lytic phages can deliver lethal or regulatory payloads to kill target bacteria without inducing lysis. This approach has been demonstrated in preclinical models targeting *Staphylococcus aureus* and *Clostridioides difficile* using CRISPR-Cas-modified phages (98–100). Temperate phages, in contrast, can be engineered to integrate therapeutic genes into resident beneficial bacteria, potentially enhancing SCFA production and Treg differentiation (101). Despite their potential, phage-based therapies face challenges, including the need to establish long-term safety, minimize off-target effects, and develop robust regulatory frameworks (101, 102).

### 5.4 Postbiotics

Postbiotics, the non-viable bacterial components or microbial metabolites, including SCFAs enzymes, peptides, and vitamins, have emerged as potential therapeutic agents with advantages in stability, safety, and manufacturing consistency compared with live microbial interventions (103). Among these, SFCAs, particularly butyrate, have been shown to exert immunomodulatory effects that are relevant across allergic diseases, including enhancement of Treg differentiation and attenuation of type 2 immune responses (104).

Evidence supporting these mechanisms derives largely from experimental models of food allergy, in which acetate and butyrate promote immune tolerance through CD103^+^ dendritic cell-dependent pathways and expansion of Treg populations (105). While food allergy and AR differ clinically, both are characterized by type 2-biased immune responses and dysregulated immune tolerance, providing a conceptual basis for considering postbiotic-mediated immunomodulation in AR. However, substantial differences in tissue context, antigen exposure, and effector mechanisms between gastrointestinal and respiratory allergy limit direct extrapolation of findings from food allergy models. Additional challenges include substantial inter-individual variability in host response, uncertainty regarding optimal dosage and delivery, and limited ability to direct postbiotic effects to specific immune pathways or tissues (103, 106).

## 6 Challenges and future perspectives in gut microbiome-based therapies for allergic rhinitis

Most microbiome-based therapeutic strategies for AR remain at the preclinical or early translational stage. While animal and *in vitro* studies have yielded valuable mechanistic insights into gut-immune-airway interactions, their translation into effective and reproducible human interventions remains challenging (107, 108). The gut microbiome represents a highly diverse and dynamic ecosystem, and substantial inter-individual variability in microbial composition, immune responsiveness, diet, age, environmental exposures and concomitant medications complicates the identification of universally effective microbial strains, consortia, or metabolites (108). These sources of biological heterogeneity necessitate stratified and context-specific therapeutic approaches rather than uniform therapeutic strategies.

Manufacturing and scalability pose additional barriers, particularly for live microbial products. Large-scale production requires stringent control over fermentation, formulation, storage, and delivery processes; all of which can influence microbial viability, stability, and functional activity *in vivo* (109). Emerging technologies such as microencapsulation, continuous fermentation systems, and rationally designed microbial consortia, may help improve product consistency and scalability, but their implementation also increases quality control and regulatory demands (109).

Regulatory consideration further constrain clinical translation and differ substantially across microbiome-based modalities (95). Live biotherapeutic products (LBP), including probiotics and FMT, are variably regulated across jurisdictions as drugs, biologics, or health products, resulting in inconsistent standards for manufacturing, quality assurance, and clinical evaluation (95, 109). Engineered microbes and bacteriophage-based therapies face additional regulatory complexity due to genetic modification, biocontainment requirements, potential horizontal gene transfer, persistence, and environmental release, for which regulatory pathways remain evolving (110). In contrast, postbiotics, as non-viable microbial products, are generally subject to less stringent oversight but still lack harmonized guidelines regarding standardization, dosing, and substantiation of therapeutic claims (103). The absence of unified, modality-specific regulatory frameworks highlights the need for clearer international guidance and early engagement with regulatory authorities during product development.

Beyond these translational and regulatory challenges, the current clinical evidence base is limited by important methodological constraints. Many studies evaluating microbiome-based interventions in AR are small and underpowered, with a high risk of publication bias favoring positive outcomes. Considerable heterogeneity exists across trials with respect to study populations, diagnostic criteria, intervention composition, dosing regimens, and treatment duration (111, 112). Clinical endpoints are often not uniform, with variable reliance on symptom scores, quality-of-life measures, and immunological markers, and limited use of validated or standardized AR-specific outcome measures (63). Follow-up periods are frequently short, restricting assessment of the durability of benefit and long-term safety (113, 114). Consequently, these factors limit cross-study comparability and weaken the strength of causal inference.

Looking forward, well-designed human clinical trials will be essential to address these gaps. Future studies should incorporate adequately powered cohorts, harmonized and validated outcome measures, and longer follow-up to assess sustained efficacy and safety, particularly in pediatric populations, as AR often manifests early in life. Integration of multi-omics approaches such as metagenomics, metabolomics, transcriptomics and immunophenotyping, along with computational tools such as artificial intelligence and machine learning, may facilitate the identification of disease-relevant microbial targets and support the development of more precise and personalized microbiome-based interventions (115).

## 7 Conclusion

Growing evidence links AR with alterations in the gut microbiome, supporting the concept that host-microbiome interactions may influence disease susceptibility and persistence. Advances in microbial profiling and biotechnology have stipulated interest in microbiome-based strategies, including probiotics, FMT, engineered microbes, and postbiotics. However, the current evidence base remains heterogeneous and is largely derived from preclinical studies or extrapolated from non-respiratory allergic disease models.

Microbial communities beyond the gut, including the nasal, oral and airway microbiome, are increasingly recognized as contributors to mucosal immune regulation. Emerging evidence suggests that gut microbial dysbiosis and gut-derived metabolites may influence immune responses at distal mucosal sites through the gut-airwa*y* axis, potentially affecting airway microbial ecology and inflammatory responses in AR. Nevertheless, the relative roles of these microbial communities in AR pathophysiology and therapeutic modulation remain incompletely defined.

Increasing attention has also been directed toward microbiome development during early life, a critical period during which immune tolerance is established and the gut microbiota transitions from a relatively simple, maternally influenced community to a more diverse, adult-like configuration. Perturbations during this developmental window have been associated with increased susceptibility to allergic diseases, prompting interest in maternal or early-life microbiome-modulating interventions aimed at shaping immune tolerance. However, the extent to which such interventions can reduce the subsequent risk of AR remains unclear.

Emerging evidence also highlights the potential contribution of non-bacterial components of the gut microbiome, including the mycobiome and virome, to host immune regulation and mucosal homeostasis. Although their roles in AR remain poorly defined, fungal and viral communities have been implicated in immune modulation and allergic inflammation, suggesting potential avenues for future therapeutic exploration (116, 117).

At present, microbiome-based approaches should be regarded as investigational or adjunctive strategies, rather than established alternatives to existing AR treatments. Their potential relevance may be greatest in selected contexts, such as early-life immune modulation, maternal-infant interventions, or as complements to conventional therapies. Future progress will depend on well-designed, adequately powered clinical trials with harmonized endpoints, longer follow-up, and clearer differentiation between microbiome modalities. Identification of reliable microbial or metabolite-based biomarkers will be essential to guide patient selection, monitor therapeutic responses, and support the development of personalized and clinically translatable microbiome-based interventions.
